# Association between tumor morphology and dosimetric parameters of organs at risk after intensity‐modulated radiotherapy in esophagus cancer

**DOI:** 10.1002/acm2.13612

**Published:** 2022-05-30

**Authors:** Fahui Li, Yuxuan Luo, Jing Chen, Liping He, Yiying Liang, Junjie Lai, Feibao Guo

**Affiliations:** ^1^ Department of Radiotherapy Cancer Center The First Affiliated Hospital of Fujian Medical University Fuzhou China; ^2^ Key Laboratory of Radiation Biology of Fujian Higher Education Institutions The First Affiliated Hospital Fujian Medical University Fuzhou China; ^3^ The Medical Technology and Engineering Academy of Fujian Medical University Fuzhou China

**Keywords:** dosimetric parameters of organs at risk, esophageal cancer, IMRT, radiation‐induced injury, tumor morphology

## Abstract

**Purpose:**

We explored the effects of geometrical topological properties of tumors such as tumor length and “axial cross‐sectional area (ACSA)” of tumors (planning target volume [PTV] volume /PTV length) on the dosimetric parameters of organs at risk (lung and heart) in patients with esophagus cancer (EPC) treated by way of intensity‐modulated radiation therapy (IMRT), so as to provide a guideline for the dosimetric limitation for organs at risk in IMRT treatment.

**Methods:**

A retrospective analysis was done on 103 cases of patients with EPC who were treated by IMRT from November 2010 to August 2019, in which PTV‐G stood for the externally expanded planning target volume (PTV) of the gross tumor volume (GTV) and PTV‐C for the externally expanded volume of the clinical target volume (CTV). A linear regression model was employed to analyze the several pairs of correlation: the 1st one between the relative length of tumors (PTV length/lung length) and pulmonary dose‐volume parameters, the 2nd one between ACSA of tumors and pulmonary dose‐volume parameters, the 3rd one between PTV length and the dosimetric parameters of the heart, and the last one between ACSA of tumors and the dosimetric parameters of the heart.

**Results:**

(i) There was a strong positive correlation between the relative length of tumors (PTV length/lung length) and *V*_5_ (*p* < 0.001, *r* = 0.73), and *V*_10_ (*p* < 0.001, *r* = 0.66) of the lung. There was a moderate positive correlation between the relative length of tumors and *V*_30_ (*p* < 0.001, *r* = 0.44) of the lung, and a weak positive correlation between the relative length of tumors and *V*_20_ (*p* < 0.001, *r* = 0.39) of the lung. (ii) There was a strong positive correlation between ACSA of tumors (PTV volume/PTV length) and *V*_30_ (*p* < 0.001, *r* = 0.67) of the lung, a moderate positive correlation between ACSA of tumors and *V*_20_ (*p* <0.001, *r* = 0.51) of the lung, and a weak positive correlation between ACSA of tumors and *V*_10_ (*p* = 0.019, *r* = 0.23) of the lung, yet there was not an obvious correlation between ACSA of tumors and *V*_5_ *p* > 0.05) of the lung. (iii) There was a moderate positive correlation between PTV length and *V*_40_ (*p* < 0.001, *r* = 0.58), and *D*_mean_ (*p* < 0.001, *r* = 0.52) of the heart, yet there was no obvious correlation between ACSA of tumors and *D*_mean_ and *V*_40_ of the heart (*p* > 0.05).

**Conclusions:**

(i) Compared with the high‐dose region of the lung, the relative length of tumors (PTV length/lung length) has a greater impact on the low‐dose region of the lung. The linear regression equation of scatter plot showed that when the relative length of tumors increased by 0.1, the lung dose‐volume parameters of *V*_5_, *V*_10_, *V*_20_, and *V*_30_ increased by approximately 5.37%, 3.59%, 1.05%, and 1.08%, respectively. When PTV length increased by 1 cm, *D*_mean_ and *V*_40_ of the heart increased by approximately 153.6 cGy and 2.03%, respectively. (ii) Compared with the low‐dose region of the lung, the value of ACSA of tumors (PTV volume/PTV length) has a greater impact on the high‐dose region of the lung. However, the value of ACSA of tumors has no significant effect on the dosimetric parameters of the heart (*D*_mean_ and *V*_40_). The linear regression equation of scatter plot showed that when ACSA of tumors increased by 10 cm^2^, the lung dose‐volume parameters of *V*_10_, *V*_20,_ and *V*_30_ increased by approximately 3.11%, 3.37%, and 4.01%, respectively.

## 1 INTRODUCTION

Esophageal cancer (EPC) is the eighth most common type of cancer in the world.^1^ Radiotherapy plays an important role in treating EPC either postoperatively or during the definitive local therapy on patients with unresectable tumors,^2^, ^3^ with its fundamental goal to maximize the therapeutic ratios (higher tumor control probability and lower normal tissue complication probability). Compared with three‐dimensional conformal radiotherapy (3D‐CRT), intensity‑modulated radiation therapy (IMRT) has better target coverage and larger potential to decrease complication rate.^4^, ^5^ However, due to the close anatomical structure of esophagus, lungs, and heart, radiotherapy for EPC will inevitably cause certain damage to the affected lungs and heart. Radiation‐induced lung and heart injury has long been considered a major restrictive factor of treatment for patients of EPC receiving thoracic radiation therapy.^6^, ^7^, ^8^ Therefore, it does matter to find a way to scientifically evaluate radiation‐induced injury of lungs and heart when making the radiotherapy planning for EPC to better prevent the occurrence of radiation‐induced injury.

In the radiotherapy of EPC, the occurrence of radiation‐induced injury of lung and heart mainly depends on two decisive factors: the patient's physical characteristics and the dosimetric parameters of organs at risk,^9^, ^10^, ^11^, ^12^, ^13^, ^14^, ^15^, ^16^ of which the latter reflects more precisely the degree of radiation‐induced injury of organs at risk in patients treated by IMRT. So a statistical analysis of the radiation dose received by organs at risk after radiotherapy is a must for a more accurate evaluation and better injury prevention. So far many researchers have tried to explore the influence of tumor volume and tumor location on the dosimetric parameters of organs at risk in radiotherapy to solve the problem mentioned earlier.^17^, ^18^ It is found that the dosimetric parameters of organs at risk (lung and heart) in patients with EPC treated by IMRT are strongly correlated with tumor location, tumor volume, and other parameters. However, few studies have been done to explore the effect of tumor morphology on the dosimetric parameters of organs at risk in the radiotherapy of EPC. It is believed that it is difficult to find a ready parameter that is fully capable of describing the specific shape of tumors, so in this paper, we defined a new parameter: “ACSA (axial cross‐sectional area)” of tumors (PTV volume/PTV length), which was combined with the parameter of tumor length to describe the geometrical topological properties of tumors. In this study, we have analyzed the effect of geometrical topological properties of tumors, mainly tumor length and ACSA of tumors (PTV volume/PTV length) on the dosimetric parameters of organs at risk (lung and heart). The variables analyzed in this study are all continuous variables. For the correlation analysis of two continuous variables, regression analysis is a commonly used statistical method. In regression, the dependent variable (the dosimetric parameters of organs at risk) is modeled as a function of independent variables (geometrical topological properties of tumors), corresponding regression parameters, and a random error. Based on the distribution trend of the scatter plot, linear regression analysis was chosen as the statistical method for this study. Therefore, a linear regression model was adopted to analyze the correlation between the geometrical topological properties of tumors and the dosimetric parameters of organs at risk (lung and heart). In this study, we try to analyze the influence of the geometrical topological properties of tumors, mainly the tumor length and ACSA of tumors, on the dosimetric parameters of organs at risk (lung and heart) in patients with EPC treated by IMRT to provide a guideline for making radiotherapy plans and preventing the occurrences of radiation‐induced injury of the lung and the heart alongside the radiotherapy.

## 2 MATERIALS AND METHODS

### 2.1 Patient population

Between 2010 and 2019, 103 patients with EPC who were undergoing IMRT in the First Affiliated Hospital of Fujian Medical University were selected as the research subjects. Patients were included only if they met the following criteria: (i) carrying pathologically proven esophageal cancer and treated with IMRT; (ii) without severe lung disease; and (iii) with detailed medical records available. In this study, only the values of tumor length and volume taken from the part between the upper and lower boundary of both lungs in the thoracic cavity were included and considered. Table 1 is the baseline characteristics of the patients selected in this research.

**TABLE 1:** Summary of the patients’ clinical characteristics

Characteristics			
Gender	Male	Number of cases 78	Percentage /% 75.7
	Female	25	24.3
Age (years)	<50	4	3.9
	50–80	84	81.6
	>80	15	14.6
Lesion location	Upper segment	24	23.3
	Middle segment	44	42.7
	Lower segment	35	34.0
PTV length (cm)	<10	7	6.8
	10‐16	67	65.0
	>16	29	28.2
PTV volume (cm^3^)	<150	7	6.8
	150‐350	72	69.9
	>350	24	23.3
Lung length(cm)	<20	17	16.5
	20‐26	72	69.9
	>26	14	13.6

Abbreviation: PTV, planning target volume.

### 2.2 Computed tomography (CT) scanning

The patients lay on the CT (AcQSim CT, Philips Medical Systems, Cleveland, USA) scanning couch and underwent scanning in the supine position with slice interval and thickness of 3–5 mm. The CT scans were performed from the first cervical spine to the lower edge of the liver and the images obtained were imported to the Raystation treatment planning system (TPS). For the cases collected in this study, four‐dimensional CT was not used in the treatment of these patients. Different from the way of observation of lung cancer, the planning target volume (PTV) of EPC is located between the two lungs, less affected by breathing movement.

### 2.3 Volume delineation

The target region and organs at risk were delineated by the radiologist in the TPS. The gross tumor volume (GTV) of EPC includes the primary and metastatic lymph node shown on CT images; and clinical target volume (CTV) includes the GTV and the area of subclinical lesions. Meanwhile, a uniform margin of 5 mm was added to the GTV and CTV to account for possible setup errors and uncertainties of positioning due to organ motion or other reasons, thus defining the planning target volume of GTV (PTV‐G) and the planning target volume of CTV (PTV‐C). Commonly, the organs at risk of EPC refer to the lung, the spinal cord, and the heart.

### 2.4 Radiotherapy planning

**FIGURE 1:**
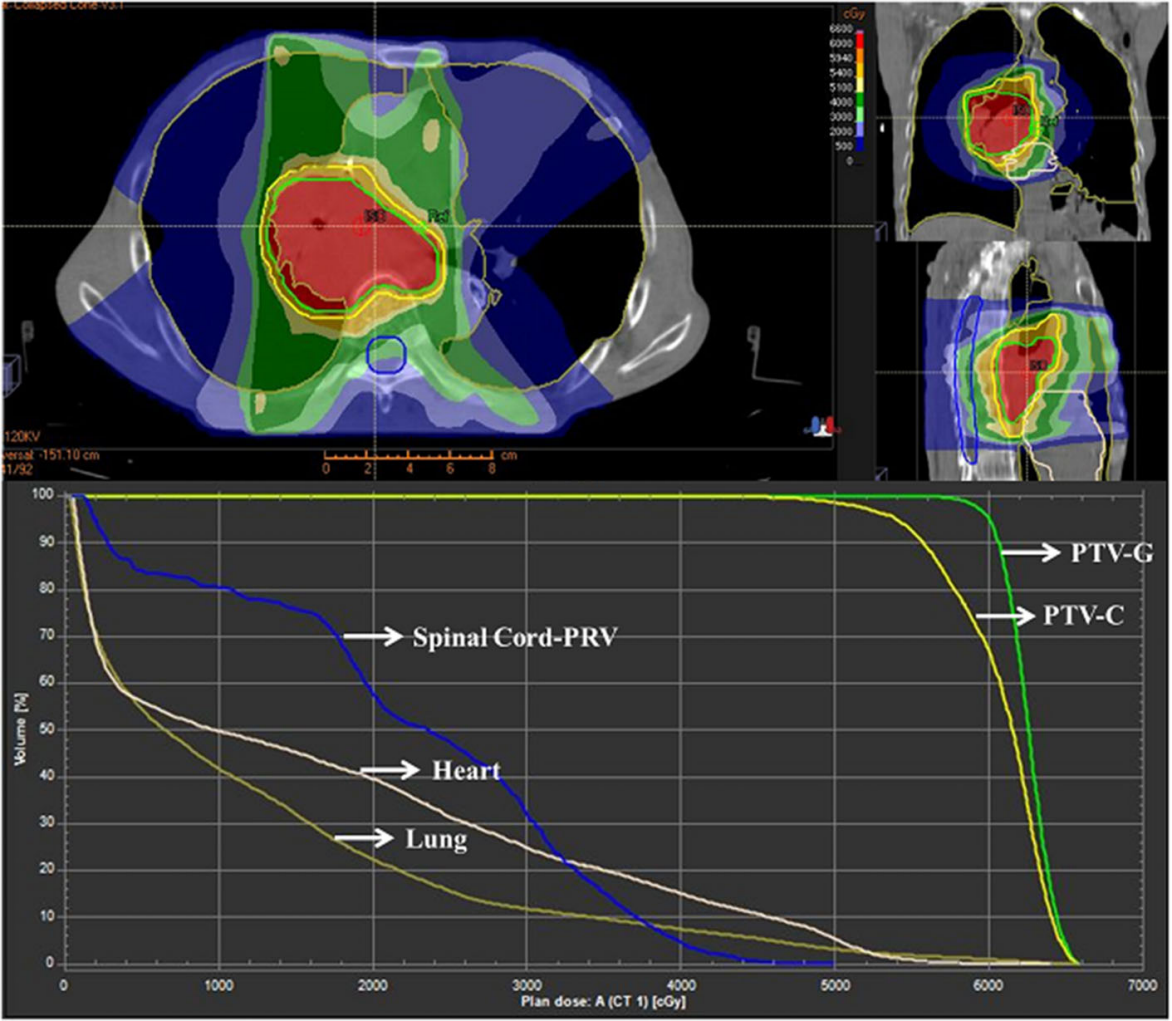
Dose distribution and dose‐volume histogram of one case with EPC in the radiotherapy treatment

In this study, a dataset of 103 patients with EPC undergoing radiotherapy was utilized. Each patient was treated with simultaneous integrated boost intensity‐modulated radiotherapy (SIB‐IMRT). The radiotherapy was performed with 6 MV X‐ray of Elekta linear accelerator. The IMRT plans to be delivered in five beams were generated by experienced dosimetrists using the Raystation TPS. The direction configuration of the 5 beams was set at 216, 300, 0, 58, and 144 degrees, respectively. The prescribed dose to the PTV‐G was 60 Gy in 2 Gy daily fractions, and the prescribed dose to the PTV‐C was 54 Gy in 1.8 Gy daily fractions. Dose constraints of surrounding organs at risk were set according to ICRU No.83 report. At least 95% of the PTV volume would be covered by the 100% prescription dose. Figure 1 is a representative radiotherapy plan including dose‐volume histogram of a patient with EPC in our study. The TPS used during the whole study was RayStation v4.7, and the collapsed cone convolution algorithm was used as the dose optimization algorithm of the TPS.

### 2.5 Record of statistical parameters

The parameters of PTV length, PTV volume, and the lung length were measured by diagnostic radiologists using axial or sagittal CT. The PTV length and the lung length are defined in the superior‐inferior direction. The dosimetric parameters of the lung (*V*_5_, *V*_10_, *V*_20_, and *V*_30_) and the heart (*D*_mean_ and *V*_40_) were calculated according to the dose volume histogram of the radiotherapy planning.

### 2.6 Statistical analysis

Statistical analysis was performed with SPSS 20.0 Package (SPSS software 20.0; SPSS, Chicago, IL, USA). The correlation between the relative length of tumors (PTV length/lung length) and the pulmonary dose‐volume parameters (*V*_5_, *V*_10_, *V*_20_, and *V*_30_) and the correlation between ACSA of tumors (PTV volume/PTV length) and the pulmonary dose‐volume parameters (*V*_5_, *V*_10_, *V*_20_, and *V*_30_) were both analyzed by an identical linear regression model. Similarly, the correlation between tumor length (PTV length) and the dosimetric parameters of the heart (*D*_mean_ and *V*_40_) and the correlation between ACSA of tumors and the dosimetric parameters of the heart (*D*_mean_ and *V*_40_) were both analyzed by another identical linear regression model.

The linear regression model is typically stated in the form *y* = *β*0 + *β*1 *x* + *ε*, where *y* is the dependent variable, *β*0 is the *y* intercept, *β*1 is the slope of the simple linear regression line, *x* is the independent variable, and *ε* is the random error. The parameters of the linear regression model need to be estimated so that the model gives the “best fit” to the data. The parameters are estimated based on predefined criterion. The most commonly used criterion is the least squares method. Whether there is a linear relationship between the independent variable and the dependent variable, that is, whether the established linear regression model is valid is the primary issue to be considered in linear regression analysis. Therefore, the hypothesis H0: *β*1 = 0 and H1: *β*1≠0 were established. The method of *t* test statistic was used to test H0: *β*1 = 0 versus H1: *β*1≠0 and a value of *p* ≤ 0.05 was considered statistically significant.

The degree of association is measured by a correlation coefficient, denoted by *r*. The correlation coefficient is measured on a scale that varies from +1 through 0 to −1. Complete correlation between two variables is expressed by either +1 or −1. When one variable increases as the other increases, the correlation is positive; when one decreases as the other increases, it is negative. Complete absence of correlation is represented by 0.

## 3 RESULTS

### 3.1 Effects of the relative tumor length and ACSA of tumors on pulmonary dose‐volume parameters

#### 3.1.1 Correlation analysis between the relative tumor length and pulmonary dose‐volume parameters

**FIGURE 2:**
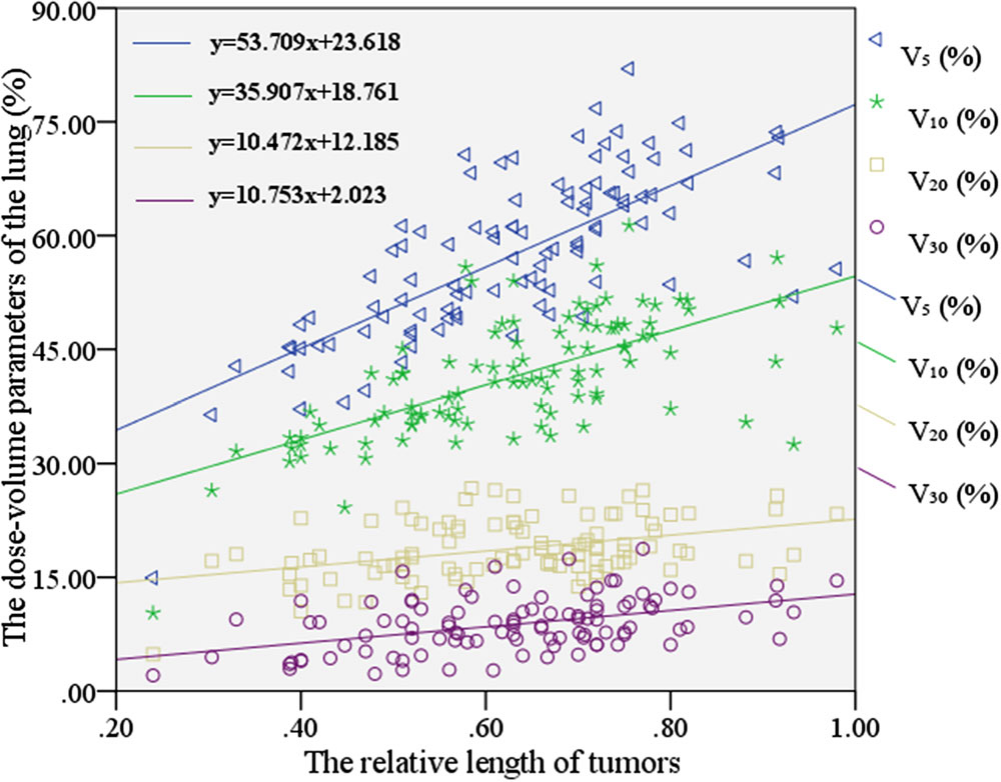
Scatter plot of the relative length of tumors (PTV length/lung length) and lung dose‐volume parameters (*V*_5_, *V*_10_, *V*_20_, and *V*_30_)

A linear regression model was used to establish the relationship between the relative length of tumors (PTV length/lung length) and the pulmonary dose‐volume parameters. As shown in Figure 2, the value of pulmonary dose‐volume parameters (*V*_5_, *V*_10_, *V*_20_, and *V*_30_) increased approximately linearly with the increase of the relative length of tumors. The statistical results (Table 2) showed that there was a strong positive correlation between the relative length of tumors (PTV length/lung length) and *V*_5_ (*p* < 0.001, *r* = 0.73) and *V*_10_ (*p* < 0.001, *r* = 0.66) of the lung. There was a moderate positive correlation between the relative length of tumors and *V*_30_ (*p* < 0.001, *r* = 0.44) of the lung, and a weak positive correlation between the relative length of tumors and *V*_20_ (*p* < 0.001, *r* = 0.39) of the lung. Compared with the high‐dose region of the lung, the relative length of tumors has a greater impact on the low‐dose region of the lung.

**TABLE 2:** Linear regression analysis results of the relative length of tumors (PTV length/lung length) and lung dose‐volume parameters in 103 EPC patients

Lung dose‐volume parameters	Correlation coefficient / *r*	*p*
*V*_5_	0.73	<0.001
*V*_10_	0.66	<0.001
*V*_20_	0.40	<0.001
*V*_30_	0.44	<0.001

#### 3.1.2 Correlation analysis of ACSA of tumors and pulmonary dose‐volume parameters

**FIGURE 3:**
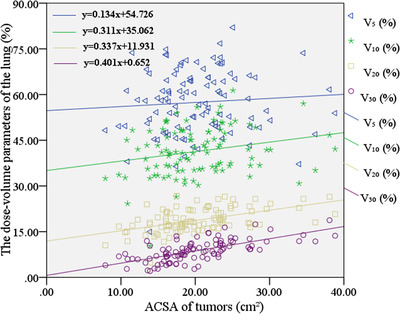
Scatter plot of ACSA of tumors (PTV volume/PTV length) and lung dose‐volume parameters (*V*_5_, *V*_10_, *V*_20_, and *V*_30_)

Another linear regression model was used to establish the relationship between ACSA of tumors (PTV volume/PTV length) and pulmonary dose‐volume parameters. As shown in Figure 3, the value of *V*_20_ and *V*_30_ of the lung increased approximately linearly with the increase of ACSA of tumors. However, the value of *V*_5_ of the lung changed nonlinearly with the increase of ACSA of tumors. The statistical results (Table 3) showed that there was a strong positive correlation between ACSA of tumors (PTV volume/PTV length) and *V*_30_ (*p* < 0.001, *r* = 0.67) of the lung, a moderate positive correlation between ACSA of tumors and *V*_20_ (*p* <0.001, *r* = 0.51) of the lung, and a weak positive correlation between ACSA of tumors and *V*_10_ (*p* = 0.019, *r* = 0.23) of the lung, yet there was not an obvious correlation between ACSA of tumors and *V*_5_ (*p* > 0.05) of the lung.

**TABLE 3:** Linear regression analysis results of ACSA of tumors (PTV volume/PTV length) and lung dose‐volume parameters in 103 EPC patients

Lung dose‐volume parameters	Correlation coefficient / *r*	*p*
*V*_5_	0.07	0.463
*V*_10_	0.23	0.019
*V*_20_	0.51	<0.001
*V*_30_	0.67	<0.001

### 3.2 Effects of tumor length and ACSA of tumors on cardiac dosimetric parameters

#### 3.2.1 Correlation analysis between PTV length and cardiac dosimetric parameters

**FIGURE 4:**
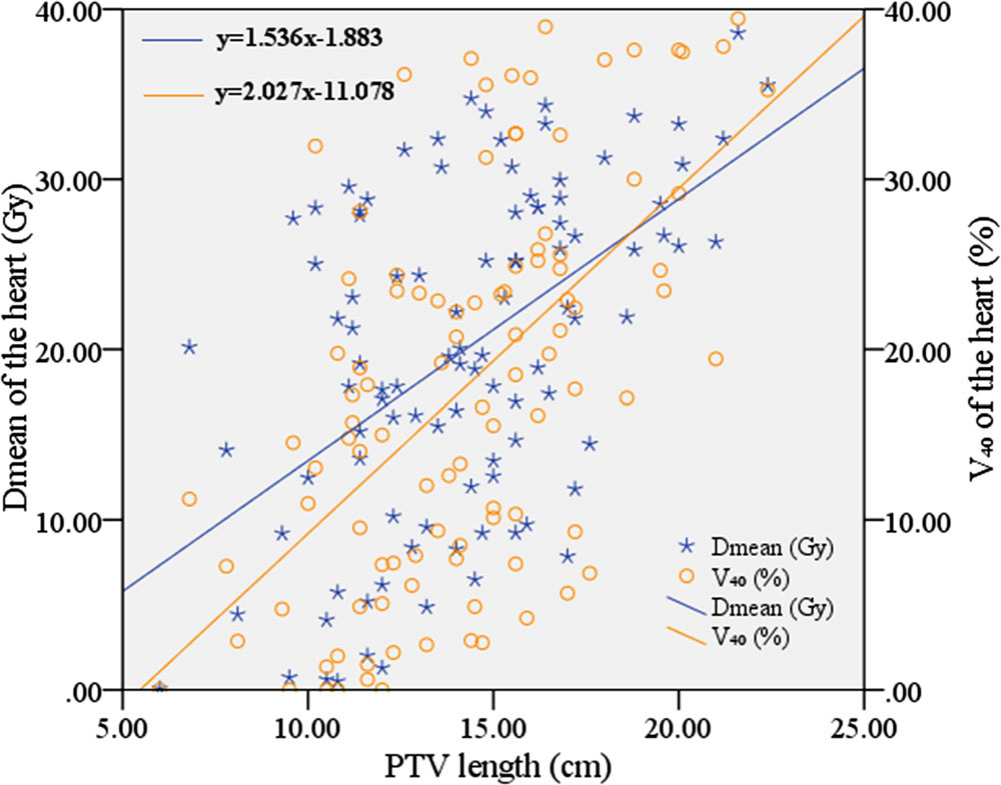
Scatter plot of tumor length (PTV length) and *D*_mean_ of the heart; Scatter plot of tumor length (PTV length) and *V*_40_ of the heart

A linear regression model was used to establish the relationship between tumor length (PTV length) and cardiac dosimetric parameters (*V*_40_ and *D*_mean_). As shown in Figure 4, the values of cardiac dosimetric parameters (*V*_40_ and *D*_mean_) increased approximately linearly with the increase of PTV length. The statistical results (Table 4) showed that, there was a moderate positive correlation between PTV length and *V*_40_ (*p* < 0.001, *r* = 0.58), and *D*_mean_ (*p* <0.001, *r* = 0.52) of the heart.

**TABLE 4:** Linear regression analysis results of tumor length (PTV length) and cardiac dosimetric parameters in 103 EPC patients

Cardiac dosimetric parameters	Correlation coefficient / *r*	*p*
*D*_mean_/Gy	0.52	<0.001
*V*_40_/%	0.58	<0.001

#### 3.2.2 Correlation analysis between ACSA of tumors and cardiac dosimetric parameters

**FIGURE 5:**
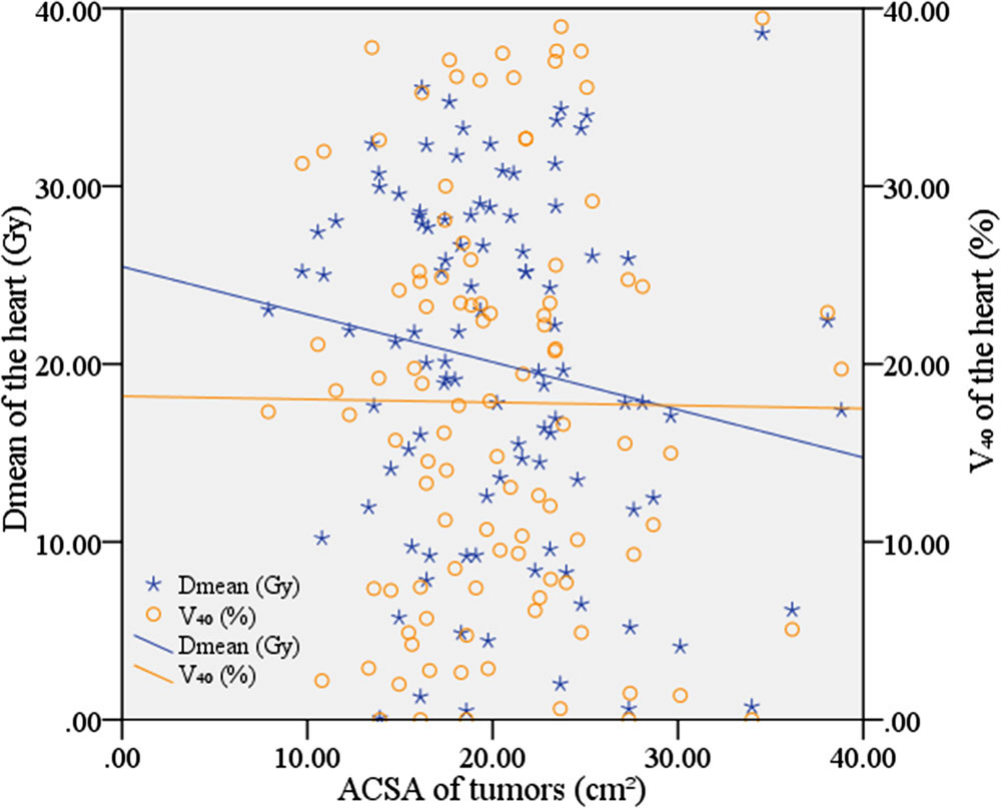
Scatter plot of ACSA of tumors (PTV volume/PTV length) and *D*_mean_ of the heart; Scatter plot of ACSA of tumors (PTV volume/PTV length) and *V*_40_ of the heart

Another linear regression model was used to establish the relationship between ACSA of tumors (PTV volume/PTV length) and cardiac dosimetric parameters (*D*_mean_ and *V*_40_). As shown in Figure 5, the value of cardiac dosimetric parameters (*D*_mean_ and *V*_40_) changed nonlinearly with the increase of ACSA of tumors. The statistical results (Table 5) showed that there was no obvious correlation between ACSA of tumors and *D*_mean_ and *V*_40_ of the heart (*p* > 0.05).

**TABLE 5:** Linear regression analysis results of ACSA of tumors (PTV volume/PTV length) and cardiac dosimetric parameters in 103 EPC patients

Cardiac dosimetric parameters	Correlation coefficient / *r*	*p*
*D*_mean_/Gy	−0.16	0.101
*V*_40_/%	0.009	0.930

## 4 DISCUSSION

Dosimetric parameters of organs at risk are important factors in evaluating the occurrence of radiation‐induced injury of organs at risk.^19^ Katsui et al.^20^ retrospectively analyzed the correlation between lung dose‐volume parameters and radiation‐induced pneumonia, indicating that the values of lung dose‐volume parameters played an important role in the prediction of radiation‐induced pneumonia. Many studies have shown that the dosimetric parameters of organs at risk (lung and heart) in patients with EPC after receiving IMRT are strongly correlated with tumor location, tumor volume, and other parameters.^17^, ^18^ However, few studies have been done to explore the effect of tumor morphology on the dosimetric parameters of organs at risk in the radiotherapy of EPC. It is believed that it is difficult to find a ready parameter to fully describe the specific shape of tumors. In this paper, we defined a new parameter: ACSA of tumors (PTV volume/PTV length), which was combined with the parameter of tumor length to describe the geometrical topological properties of tumors. Further, the correlation between the geometrical topological properties of tumors (mainly tumor length and ACSA of tumors) and the dosimetric parameters of organs at risk (lung and heart) was analyzed with a linear regression model.

The ratio of PTV length to lung length represents the relative length of tumors against the length of the lung. Theoretically, with the increase of the relative length of tumors (PTV length/lung length), the values of lung dose‐volume parameters become larger along. And the ratio of PTV volume to PTV length represents the ACSA of tumors. In this study, tumor length and ACSA of tumors were used to describe the geometrical topological properties of tumors. From the linear regression equation of scatter diagram and the statistical results, we can approximately evaluate how tumor morphology affects the dosimetric parameters of the lung and the heart. The results from the study discussed in this paper showed that: (i) There was a strong positive correlation between the relative length of tumors (PTV length/lung length) and *V*_5_ (*p* < 0.001, *r* = 0.73), and *V*_10_ (*p* < 0.001, *r* = 0.66) of the lung. There was a moderate positive correlation between the relative length of tumors and *V*_30_ (*p* < 0.001, *r* = 0.44) of the lung, and a weak positive correlation between the relative length of tumors and *V*_20_ (*p* <0.001, *r* = 0.39) of the lung. As shown in Figure 2, the value of pulmonary dose‐volume parameters (*V*_5_, *V*_10_, *V*_20_, and *V*_30_) increased approximately linearly with the increase of the relative length of tumors. The linear regression equation of scatter plot (Figure 2) showed that when the relative length of tumors increased by 0.1, the lung dose‐volume parameters of *V*_5_, *V*_10_, *V*_20_, and *V*_30_ increased by approximately 5.37%, 3.59%, 1.05%, and 1.08%, respectively. Compared with the high‐dose region of the lung, the relative length of tumors has a greater impact on the low‐dose region of the lung. (ii) There was a strong positive correlation between ACSA of tumors (PTV volume/PTV length) and *V*_30_ (*p* < 0.001, *r* = 0.67) of the lung, a moderate positive correlation between ACSA of tumors and *V*_20_ (*p* <0.001, *r* = 0.51) of the lung, and a weak positive correlation between ACSA of tumors and *V*_10_ (*p* = 0.019, *r* = 0.23) of the lung, yet there was not an obvious correlation between ACSA of tumors and *V*_5_ (*p* > 0.05) of the lung. As shown in Figure 3, the value of *V*_20_ and *V*_30_ of the lung increased approximately linearly with the increase of ACSA of tumors. However, the value of *V*_5_ of the lung changed nonlinearly with the increase of ACSA of tumors. The linear regression equation of scatter plot (Figure 3) showed that when ACSA of tumors increased by 10 cm^2^, the lung dose‐volume parameters of *V*_10_, *V*_20_, and *V*_30_ increased by approximately 3.11%, 3.37%, and 4.01%, respectively. In conclusion, compared with the low‐dose region of the lung, ACSA of tumors had a greater impact on the high‐dose region of the lung. (iii) There was a moderate positive correlation between PTV length and *V*_40_ (*p* <0.001, *r* = 0.58), and *D*_mean_ (*p* < 0.001, *r* = 0.52) of the heart. As shown in Figure 4, the values of cardiac dosimetric parameters (*V*_40_ and *D*_mean_) increased approximately linearly with the increase of PTV length. The linear regression equation of scatter plot (Figure 4) showed that when PTV length increased by 1 cm, *D*_mean_ and *V*_40_ of the heart increased by approximately 153.6 cGy and 2.0%, respectively. (iv) There was not an obvious correlation between ACSA of tumors (PTV volume/PTV length) and *V*_40,_ and *D*_mean_ of the heart (*p* > 0.05). As shown in Figure 5, the value of cardiac dosimetric parameters (*D*_mean_ and *V*_40_) changed nonlinearly with the increase of ACSA of tumors. Such results elaborated earlier provided the following guidelines for clinical physicists to formulate radiotherapy plans for the patients with EPC: (i) for EPC patients with large tumor length, more attention should be paid to the dosimetry limits of the lung and the heart when IMRT plan is being designed, especially for the low‐dose region of the lung; and (ii) for EPC patients with large ACSA of tumors, more attention should be paid to the limits of high‐dose region of the lung, so as to reduce the occurrence of irradiation‐induced injury.

It can be seen from the results that the relative length of tumors (PTV length/lung length) has a greater impact on the low‐dose region of the lung, while ACSA of tumors (PTV volume/PTV length) has a greater impact on the high‐dose region of the lung. On the other hand, the value of ACSA of tumors has no significant effect on the dosimetric parameters of the heart. The reasons may be as follows: (i) In this study, only the values of tumor length and tumor volume measured at the part between the upper and lower boundary of both lungs in the thoracic cavity were taken and considered. As the relative length of tumors increases, more lung tissue is affected by the radiation dose, resulting in an increase in the value of the lung dose‐volume parameters, especially for *V*_5_ and *V*_10_ of the lung. There is a certain distance between the two lungs, and the high‐dose region of the lung is located adjacent to the tumor. Therefore, compared with the low‐dose region of the lung, the increase in the relative length of tumors has less effect on the high‐dose region of the lung. (ii) The larger the ACSA of tumors is, the closer the tumor is to both lungs, resulting in the increase of the lung tissue adjacent to the tumor and eventually leading to the increase of the high‐dose region of the lung. Compared with the high‐dose region of the lung, the low‐dose region of the lung is farther away from the tumor. Therefore, the low‐dose region of the lung is less affected by ACSA of tumors. (iii) Tumors of EPC are located between the two lungs. Both lungs are greatly affected by ACSA of tumors. However, due to the uncertainty of the relative position between tumors of EPC and the heart, ACSA of tumors has no significant effect on cardiac dosimetry parameters.

## 5 CONCLUSION

Previous studies were mostly focused on the correlation between lung dose‐volume parameters and radiation‐induced pneumonia, indicating that the values of lung dose‐volume parameters played an important role in the prediction of radiation‐induced pneumonia.^9^, ^10^, ^11^, ^12^, ^13^, ^14^, ^15^, ^16^ In this study, we have analyzed the effect of geometrical topological properties of tumors, mainly tumor length and ACSA of tumors (PTV volume/PTV length) on the dosimetric parameters of organs at risk (lung and heart), so as to provide a guideline for the dosimetric limitation of organs at risk in IMRT treatment of EPC before a radiotherapy plan is made. The results of this study have shown that: (i) Compared with the high‐dose region of the lung, the relative length of tumors (PTV length/lung length) has a greater impact on the low‐dose region of the lung. The linear regression equation of scatter plot showed that when the relative length of tumors increased by 0.1, the lung dose‐volume parameters of *V*_5_, *V*_10_, *V*_20_, and *V*_30_ increased by approximately 5.37%, 3.59%, 1.05%, and 1.08%, respectively. When PTV length increased by 1 cm, *D*_mean_ and *V*_40_ of the heart increased by approximately 153.6 cGy and 2.03%, respectively. (ii) Compared with the low‐dose region of the lung, the value of ACSA of tumors (PTV volume/PTV length) has a greater impact on the high‐dose region of the lung. However, the value of ACSA of tumors has no significant effect on the dosimetric parameters of the heart (*D*_mean_ and *V*_40_). The linear regression equation of scatter plot showed that when ACSA of tumors increased by 10 cm^2^, the lung dose‐volume parameters of *V*_10_, *V*_20_, and *V*_30_ increased by approximately 3.11%, 3.37%, and 4.01%, respectively. Although in this article, the research is still of preliminary stage, the correlation between geometrical topological properties of tumors and the dosimetric parameters of organs at risk (lung and heart) in the radiotherapy of EPC could be proved. The next step of this research is to build the relationship between multiparameters of EPC (including tumor relative location, tumor volume, and geometrical topological properties of tumors) and the dosimetric parameters of organs at risk (lung and heart), so as to predict precisely the dosimetric parameters of organs at risk in the radiotherapy of EPC, in the hope that by introducing the parameter of geometrical topological properties of tumors, the therapists could evidently improve the prediction accuracy and obtain a guideline for clearly defining the dosimetric limitation of organs at risk in IMRT treatment of EPC.

## CONFLICT OF INTEREST

The authors declare no conflict of interest.

## AUTHOR CONTRIBUTIONS

*Study concept/study design*: Feibao Guo and Fahui Li. *Methodology*: Fahui. *Statistical analysis and manuscript writing*: Fahui Li. *Data acquisition*: Yuxuan Luo, Jing Chen, Liping He, Yiying Liang, and Junjie Lai.

## Data Availability

The data that support the findings of this study are available from the corresponding author upon reasonable request.
